# Influencing factors of self-regulated learning of medical-related students in a traditional Chinese medical university: a cross-sectional study

**DOI:** 10.1186/s12909-023-04051-4

**Published:** 2023-02-03

**Authors:** Ling Li, Ming-ling Zhu, Yu-qing Shi, Li-li Yang

**Affiliations:** 1 School of Nursing, Zhejiang Shuren University, 8 Shuren Road, Hangzhou, ZheJiang 310015 People’s Republic of China; 2 School of Nursing, Zhejiang Chinese Medical University, 548 Bin-Wen Road, Hangzhou, Zhejiang 310053 People’s Republic of China

**Keywords:** Medical-related students, Self-regulated learning, Learning strategy medical education

## Abstract

**Background:**

In recent years, self-regulated learning (SRL) has become a hot topic in medical education. However, the factors that affect the SRL ability of medical-related specialties, such as clinical medicine, traditional Chinese medicine (TCM), and nursing specialty in TCM colleges and universities are unclear. Whether the teaching of learning strategies can help improve students’ SRL also needs to be further examined.

**Method:**

A cross-sectional survey was distributed, and 878 medical-related students who were from a TCM university were recruited for this study. Descriptive statistics illustrated the status quo of SRL and learning strategies, and an independent t-test and analysis of variance were used to analyze the factors associated with SRL. The relationship between SRL and learning strategies was analyzed with multi-linear regression analysis.

**Results:**

The scores of SRL on learning motivation, learning setting, self-regulation, and total scores were 34.76 ± 4.62, 41.14 ± 4.30, 39.26 ± 4.74, and 115.16 ± 12.42, respectively. The metacognitive, emotion, cognitive, resource management and total scores of learning strategies were 58.54 ± 12.02, 43.24 ± 8.42, 35.49 ± 7.34, 22.89 ± 4.20, 160.16 ± 29.45, and the mean was all above the midpoint. Learning strategies were positively correlated with SRL (*r* = 0.421, *P* < 0.01). Some factors can predict 32% of the variation of SRL, including whether they liked their specialty, educational system, specialty, score ranking, scholarship, whether they were taught by a tutor in middle school, gender, monthly family income, the father's educational background, metacognitive strategy, resource management strategy, and cognitive strategy.

**Conclusions:**

The SRL of medical-related students was better. Learning strategies, as well as personal or social factors, can affect SRL. Educators should pay more attention to the cultivation of learning strategies, exercising learning skills, and monitoring, adjustment, and guidance of learning time. It should adopt various methods to improve the SRL of medical-related students according to the different factors.

## Background

Under China’s background as a learning society and its acceleration of the construction of an innovative country, one trend is to establish the concept of lifelong learning. The high demands of the medical profession require lifelong learning and moral development, which should be a focus during medical education.

Self-direction and self-regulation are two parts of lifelong learning. Self-regulated learning (SRL) consists of three stages. The first is anticipation before knowledge, which includes task analysis and goal setting. The second is performance control, which involves using cognitive strategies, such as rehearsal, elaboration, organization, and metacognitive monitoring. The third point is self-reflection, in which students judge and develop the reasons for their learning behavior. This process is dynamic and cyclic [1], active and constructive [2], and is the result of which learners decide to obtain the information they want to experience or learn [3]. It is also believed that SRL is a process of metacognition, motivation, and dynamic behavior in the learning [4], which involves four major elements: planning, learning, self-assessment, and monitoring. Given that individuals are guided and constrained by the background, characteristics of the target, and the environment, the SRL ability of individuals is vital to the formation of lifelong learning [2]. Doctors are all expected to continually improve their skills and keep pace with developments in their field. Thus, they need to become lifelong learners, and promote the development of their SRL greatly [5]. Medical students need to control their learning process [1, 6] while also handling more ‘high-risk’ tasks such as testing, interviewing, and preparing for work, and requiring specific SRL skills to manage their learning behavior, motivation and emotions effectively [7]. The goal of medical education is to develop doctors who can become lifelong learners [8]. Studies have found that clinical medical students’ SRL is influenced by individual, background, social factors, and autonomic experience [9], as well as by other variables such as gender, educational environment, teaching methods, personality, and family background [10]. A higher level of SRL is positively correlated with academic performance and clinical skills [11–15] and negatively associated with depression [16]. SRL is a skill that students learn, not acquired naturally, that can be learned and therefore taught [17]. In scaffolding of instructional guidance, the cognitive apprenticeship approach may support SRL; blended learning drives students’ motivation and SRL [18].

Learning strategy refers to an operating system in which learners actively operate learning procedures, tools, and methods effectively in a particular context, according to specific learning tasks and general learning rules to improve the quality and efficiency of learning. It is a multi-level and dynamic operating system, comprising four parts. Cognitive strategies include retelling, finishing, and organizing. Metacognitive strategies include planning, monitoring, and execution strategies. Emotional strategy are related to learning motivation, interest, and attitude. Resource management strategies include time management, learning environment management, and help-seeking [19]. Learners of SRL activate, maintain, and adjust their cognition, emotion, and action purposefully. They need to adopt different learning strategies to achieve their learning goals. Such methods include being able to set their learning goals, establish a more productive environment, monitor their understanding, and modify their plans, strategies, and efforts in response to changing environmental conditions [20]. Mastering specific learning strategies is necessary for good SRL. Students learning strategies have a significant impact on their academic performance. A study has found that learning strategies are positively correlated with SRL [21]. Learning strategies help integrate knowledge and activate experience and are of great theoretical and practical significance for promoting effective learning and realizing educational and teaching objectives in universities [22].

Medical education in China is divided into two main categories, clinical medicine and traditional Chinese medicine namely, among them, traditional Chinese medical colleges focus on training talents in traditional Chinese medicine. With the surge in China's population, economic development, and improvement of living standards, the demand for quality of life and healthcare workers has also increased. Nowadays, traditional Chinese medical colleges have also set up clinical medicine, nursing, etc. At present, traditional Chinese medical colleges and universities have a five-year program in undergraduate education of traditional Chinese medicine and clinical medicine, four-year program in undergraduate nursing, a three-year program in junior nursing in adult teaching schools, and postgraduate training in corresponding specialties. Both traditional Chinese medicine and clinical medicine are composed of introductory medical courses and clinical medical courses to the fifth year of internship. At the same time, the undergraduate nursing program is four years, which courses comprised of basic medical and nursing courses, and clinical nursing courses until the fourth year internship, get a Bachelor of Science degree when they graduate. The particularity of the medical profession requires medical students to have excellent qualities, such as exquisite knowledge, profcient skills, and moral character. Improving SRL and lifelong learning ability is an inevitable choice for their career development [23]. However, many medical students adopt unsuccessful learning strategies and study habits. Students with poor time management strategies might get more unsatisfactory academic performance because they often assume learning techniques such as cramming and surface learning [24]. A total of 49% of the variance in examination scores are accounted for by the preferred learning strategy of students who are in a pre-clinical medical period [25]. Due to the lack of clinical knowledge combined with introductory courses in the early stage of medical students, students will inevitably feel unimaginative in the learning process, and their learning interest decreases gradually. The “cramming” teaching mode is still used by many traditional Chinese medical colleges, which is extremely dull, and also affects students’ interest in learning [26]. Secondly, the student’s learning mainly depends on teachers’ knowledge teaching, which affects the development of their SRL due to the influence of exam-oriented education in China. There are some characteristics of SRL among traditional Chinese medical college students, such as weak consciousness of SRL, unitary structure and way of learning knowledge, over-exaggeration of students, SRL, lack of supervision and management of teachers, and inadequate professional guidance [27]. Nowadays, online learning and blended learning have become popular trends. The medical-related students in traditional Chinese medical universities need to master the knowledge, including traditional Chinese medicine and clinical medical science. They will carry out heavy tasks in a limited period, and the demand for grasping learning strategies and SRL ability seems to be more critical.

The significance of this study is to explore the status quo and influencing factors of SRL among medical-related students in traditional Chinese medical universities and to provide an evidence basis for intervention measures to cultivate better the SRL of medical-related students in higher medical education. Based on the previous literature review, the following research questions are presented

1. What is the status quo of SRL for medical-related students in TCM colleges and universities?
2. Does a relationship exist between the SRL and the learning strategy of medical-related students?
3. What are the factors influencing of medical-related students’ SRL in a traditional Chinese medical university?
4. How can the SRL of medical-related students in TCM colleges and universities be improved?

## Method

### Participants and procedures

The study was conducted from March 2020–May 2021 at one university in China. A cross-sectional design was adopted. A total of 878 medical-related students comprising 683 females and 195 males, were recruited using the convenient sampling method. We collected the data from the samples which were first-, second-, third-, fourth- and fifth-year students, including those were majoring in nursing in nursing college(406 people in total), TCM in the first clinical medical college (167 people), clinical medicine in the second clinical medical college (202 people), and nursing in the extended education nursing college (103 people) using structured questionnaires. The inclusion criteria were as follows. Medical and nursing students studying at a TCM university frmo March 2020–May 2021 were willing to participate in this study. Part-time medical-related students were omitted, which is our exclusion criteria. The survey was conducted in Chinese, and online inquiry was conducted through the Questionnaire network, the survey platform. Surveys were distributed only to those who consented to study participation directly. All participants were informed of the details of the study’s purpose before the survey was administered, and informed that data collected in this survey would be aggregated and published. Their identities and name information have not been included in this study. The survey objects would fill in independently for about 30 min.

### Instruments

#### Demographic questionnaire

The demographic characteristics of the participants included gender, age, specialty (traditional medical medicine, clinical medicine, and nursing), educational system, subjects before admission (liberal arts, science), the only child or not, whether got a scholarship, availing of student loans, admission batch (the second batch, public, which means that the students with higher scores in the college entrance examination, and they fit with the standards and proportion of the Ministry of Education’s undergraduate teaching admission, so their tuition is lower. The third batch, is private, meaning that their college entrance examination just missed the threshold for undergraduate course admission, so the Ministry of Education permits them to enter colleges with undergraduate education teaching standards. They do not belong to the security group invested by the Ministry of Education, so their tuition is higher. Others, this refers to adult education), grade, monthly family income, the parent’s educational background, whether they were taught by a tutor in middle school or not, student cadre, score ranking, whether they like their specialty or not, and whether they are in love or not.

#### College Student SRL Questionnaire

The College Student SRL Questionnaire was developed by Wang Xian Liang [28] in 2006. It has 33 items in total. A five-point Likert scale was adopted, in which ‘very consistent,’ ‘consistent,’ ‘sometimes consistent,’ ‘inconsistent’, and ‘very inconsistent’ were recorded as 5, 4, 3, 2, and 1, respectively. The total scores of SRL ranged from 33 to 165, and higher scores mean more substantial SRL ability. The Cronbach’s α of the learning motivation, learning settings, and self-adjustment subscale were 0.731, 0.821, and 0.754, respectively, and the Cronbach’s α of the total scale was 0.899. The contribution rates of the three subscales of learning motivation, learning settings, and self-adjustment were 56.573%, 51.872%, and 53.289%, respectively. The total cumulative contribution rate of the three subscales was 78.887%, which had good reliability and validity. The Cronbach’s α = 0.922 for this study scale. The Cronbach’s α of learning motivation, learning settings, and self-adjustment subscales were 0.832, 0.779, and 0.810, respectively.

#### College student learning strategy questionnaire

Developed by Yang Yi in 2002 [29], the questionnaire has 49 questions, comprising four subscales: metacognitive strategy, emotional strategy, cognitive strategy, and resource management strategy. The self-rating scale uses a five-point Likert rating scale in which 1, 2, 3, 4, and 5, were successively used for ‘complete non-conformity,’ ‘quite inconsistent,’ ‘not clear,’ ‘more conformity,’ and ‘complete conformity.’ The higher the score, the more learning strategies are used. The factor analysis during the development of this scale verified the theoretical construction, and the correlation coefficient between the subscales and the total scale was between 0.604–0.937. The correlation coefficient between students’ academic performance and the learning strategy questionnaire was taken as the criterion validity. The correlation coefficient between the four subscales and the calibration standard was 0.722–0.896. The Cronbach’s α of the metacognitive strategy, emotional strategy, cognitive strategy, and resource management strategy subscale was 0.884, 0.772, 0.774, and 0.689, respectively. The Cronbach’s α of the total scale was 0.9332. The Cronbach’s α was 0.972 for this study scale. The Cronbach’s α of metacognitive strategy, emotional strategy, cognitive strategy, and resource management strategy were 0.946, 0.912, 0.902, and 0.825, respectively.

### Ethical approval

This study was approved by the ethical Committee of Zhejiang Shu Ren University and complied with the code of the Declaration of Helsinki (1964) and its later amendments. Medical students were assured that participation was voluntary and they could withdraw anytime if they wanted to without any punishment. Students were told that all data was obtained from the survey.

### Statistical analysis

The data were analyzed using SPSS version 25.0 (IBM, Beijing, China) for Windows. Categorical variables were expressed as numbers and percentages, and continuous variables were expressed as mean ± standard deviation (SD). A descriptive statistical method was applied regarding the characteristics of the sample and research variables. We also tested that normal distribution of the data in two or more groups was satisfactory, variance inflation factor (VIF) was used to examine the multicollinearity of the regression analysis. Durbin-Watson (DW) autocorrelation statistic was generated to identify models with serial autocorrelation. In this study, the values of VIF (1.066–4.886) and DW (1.685) were both in the allowable range [30]. An independent t-test for independent models and one-way ANOVA statistical method were used to compare the SRL in terms of the categorical socio-demographic characteristics. Pearson correlation analyses were conducted to perceive the associations between all continuous variables. To assess the influence of different factors on SRL, we performed a multiple linear regression analysis with the total score of SRL as dependent variable. The significant variables in the results of the univariate analysis were independent variables, and the forward stepwise method was used. A *p*-value of < 0.05 and *P* < 0.01 were considered statistically significant.

## Results

### Descriptive statistics of the SRL and learning strategies

A total of 910 questionnaires were issued, all of which were recovered, for a 100% recovery rate. Thirty-two questionnaires were ineffective (which had toomany differences between the average answer time, and the choices presented some patterns, such as zigzag answers), resulting in 878 effective questionnaires, with an effective rate of 96.48%. The demographic characteristics were as follows. For gender, 683 (77.79%) were female. For specialty, 509 (57.97%) were nursing, 167 (19.02%) were TCM, and clinical medicine was 202 (23.01%). The educational system was four years 406 (46.24%), and five years 369 (42.03%). For subjects before admission, 623 (70.96%) respondents studied science. For only child status, 489 (55.69%) answered no. For scholarships, 334 (38.04%) were accepted. For student loans, 113 (12.87%) were availed. For admitted batch, 290 (33.03%) were in the second batch, and 464 (52.85%) were in the third batch. For grades, the majority were in third grade, which was 446 (50.80%). For family income, 60 (6.83%) earned less than CNY2000, 180 (20.50%) were CNY2001–5000, 252 (28.70%) were CNY 5001–8000, 162 (18.45%) were CNY 8001–10,000, 224 (25.51%) were above CNY 10,000. For the father’s educational background, the middle school had a 47.04% proportion (413). Mothers shared the same educational background as fathers. 375 (42.71%) respondents were in a student cadre. A total of 269(30.64%) employed a tutor in high school. For score ranking, 185 (21.07%) were upper, 523 (59.57%) were middle, and 170 (19.36%) were lower. 262 (29.84%) liked his specialty, and 36 (4.10%) disliked them. A total of 265 (30.18%) were in love. The demographic characteristics are all shown in Table 1.

**Table 1:** Summary statistics for key variables (*N* = 878)

	**Participants (N)**	**% of total N**
**Gender**		
Female	683	77.79
Male	195	22.21
**Specialty**		
traditional Chinese medicine	167	19.02
Clinical medicine	202	23.01
Nursing	509	57.97
**Educational system**		
3 years	103	11.73
4 years	406	46.24
5 years	369	42.03
**Divide subjects before admission**		
liberal arts	255	29.04
science	623	70.96
**The only child**		
yes	389	44.31
no	489	55.69
**Scholarship**		
yes	334	38.04
no	544	61.96
**Student loan**		
yes	113	12.87
no	765	87.13
**Admission batch**		
Second (public)	290	33.03
Third (private)	464	52.85
others	124	14.12
**Grades**		
1^st^ grade	253	28.82
2^nd^ grade	128	14.58
3^rd^ grade	446	50.80
4^th^ grade	51	5.81
**Monthly family income**		
Below CNY 2000	60	6.83
CNY 2001–5000	180	20.50
CNY 5001–8000	252	28.70
CNY8001-10,000	162	18.45
CNY 10,000 above	224	25.51
**The father’s educational background**		
Primary school	143	16.29
Middle school	413	47.04
High school	228	25.97
University	82	9.34
University above	12	1.37
**The mother’s educational background**		
Primary school	217	24.72
Middle school	399	45.44
High school	212	24.15
University	43	4.90
University above	7	0.80
**Student cadres**		
yes	375	42.71
no	503	57.29
**Whether be taught by a tutor in middle school or not**		
yes	269	30.64
no	609	69.36
**Score ranking**		
upstream	185	21.07
midstream	523	59.57
downstream	170	19.36
**Whether like his specialty or not**		
yes	262	29.84
commonly	580	66.06
no	36	4.10
**In love**		
yes	265	30.18
no	613	69.82

In the univariate factor analysis shown in Table 2, there were significant differences in SRL scores between different groups (*p* < 0.05). Compared with the females, the male students got higher SRL scores. And the clinical medical students had the highest scores than traditional Chinese medical science and nursing, the students whose educational system is 3-years had the lowest scores, and those who were divided into science subjects before admission had better SRL. As an only child in the family, the students’ SRL scores were higher, those who had not got a scholarship or accepted adult education, and studied in 1^st^ and 2^nd^ grades had the lower SRL. Students whose parents are highly educated, have good family income, and can hire tutors in high school had higher SRL scores. Those who were student cadres, and preferred their specialty also had better SRL. The higher the score ranking, the better the SRL.

**Table 2:** Univariate analysis and post hoc test on influencing factors of Self-regulated Learning (*n* = 878)

Variables	Items	N(%)	Self-regulated	
			**Learning**	
			**Mean ± SD**	**p**
				**LSD**
Gender	female	683(77.79)	114.45 ± 11.63	**0.010**
	male	195(22.21)	117.65 ± 14.62	
	nursing	509(57.97)	113.76 ± 12.24	
Specialty	traditional Chinese medical science	167(19.02)	115.72 ± 11.74	**0.000** **(2 > 1,3)**
	clinical medicine	202(23.01)	118.75 ± 13.05	
	nursing	509(57.97)	113.76 ± 12.24	
Educational	3 years	103(11.73)	108.12 ± 11.26	**0.000** **(1 < 2 < 3)**
system	4 years	406(46.24)	115.19 ± 12.07	
	5 years	369(42.03)	117.09 ± 12.43	
Divide subjects	lliberal arts	255(29.04)	113.31 ± 12.37	**0.010**
before admission	science	623(70.96)	115.92 ± 12.37	
The only child	yes	389(44.31)	116.23 ± 11.72	**0.020**
	no	489(55.69)	114.31 ± 12.90	
Scholarship	yes	334(38.04)	118.97 ± 13.50	**0.000**
	no	544(61.96)	112.82 ± 11.09	
Student loan	yes	113(12.87)	114.07 ± 12.94	0.320
	no	765(87.13)	115.32 ± 12.34	
Admission batch	Second (public)	290(33.03)	115.61 ± 10.86	**0.000** **(3 < 1,2)**
	Third (private)	464(52.85)	116.64 ± 13.08	
	others	124(14.12)	108.59 ± 11.24	
Grades	1^st^ grade	253(28.82)	112.87 ± 12.77	**0.000** **(1,2 < 3,4)**
	2^nd^ grade	128(14.58)	112.65 ± 9.81	
	3^rd^ grade	446(50.80)	116.85 ± 12.59	
	4^th^ grade	51(5.81)	118.09 ± 12.42	
Monthly family income	Below CNY 2000	60(6.83)	114.58 ± 13.12	**0.000** **(5,4 > 1,3 > 2)**
	CNY 2001–5000	180(20.50)	111.9 ± 11.83	
	CNY 5001–8000	252(28.70)	114.42 ± 11.91	
	CNY8001-10,000	162(18.45)	116.49 ± 11.63	
	CNY 10,000 above	224(25.51)	117.82 ± 12.42	
The father’s educational background	Primary school	143(16.29)	113.27 ± 13.29	**0.010** **(5 > 1,2,3,4)**
	Middle school	413(47.04)	115.66 ± 12.22	
	High school	228(25.97)	115.96 ± 12.12	
	University	82(9.34)	112.55 ± 12.22	
	University above	12(1.37)	123.17 ± 10.22	
The mother’s educational background	Primary school	217(24.72)	112.94 ± 12.79	**0.000** **(1 < 2,3 < 4 < 5)**
	Middle school	399(45.44)	115.66 ± 12.06	
	High school	212(24.15)	115.30 ± 12.65	
	University	43(4.90)	119.63 ± 11.18	
	University above	7(0.80)	124.29 ± 8.81	
Student cadres	yes	375(42.71)	117.28 ± 12.08	**0.000**
	no	503(57.29)	113.58 ± 12.44	
Whether be taught by a tutor in middle school or not	yes	269(30.64)	117.98 ± 13.45	**0.000**
	no	609(69.36)	113.92 ± 11.73	
Score ranking	upstream	185(21.07)	121.49 ± 13.00	**0.000** **(1 > 2 > 3)**
	midstream	523(59.57)	114.27 ± 11.61	
	downstream	170(19.36)	111.02 ± 11.68	
Whether like his specialty or not	yes	262(29.84)	120.5 ± 13.10	**0.000** **(1 > 2,3)**
	commonly	580(66.06)	113.02 ± 11.49	
	no	36(4.10)	110.78 ± 9.57	
In love	yes	265(30.18)	115.96 ± 13.35	0.210
	no	613(69.82)	114.82 ± 11.99	

Statistically significant values are indicated in bold. The differences and order among groups in the post-hoc comparison are indicated by superscript letters (1,2,3,4,5)

Table 3 shows the means, standard deviations, and Pearson correlation analysis of continuous variables. The results showed that there was a significant correlation between the variables. The results indicated that medical students’ learning strategy (Mean = 160.16), and SRL (Mean = 115.16) were above the median. There was a good correlation between them (*r* = 0.421, *P* < 0.01).

**Table 3:** Means, standard deviation (SD), and correlations of continuous variables

	**Mean**	**SD**	**1**	**2**	**3**	**4**	**5**	**6**	**7**	**8**	**9**
**1Learning Strategy**	160.16	29.45	1								
2Metacognitive strategy	58.54	12.02	0.965^**^	1							
3Emotional strategy	43.24	8.42	0.938^**^	0.858^**^	1						
4Cognitive strategy	35.49	7.34	0.922^**^	0.860^**^	0.830^**^	1					
5Resource management strategy	22.89	4.20	0.759^**^	0.680^**^	0.668^**^	0.593^**^	1				
**6Self-regulated Learning**	115.16	12.42	0.421^**^	0.432^**^	0.372^**^	0.334^**^	0.389^**^	1			
7learning motivation	34.76	4.62	0.362^**^	0.376^**^	0.304^**^	0.277^**^	0.368^**^	0.925^**^	1		
8Learning settings	41.14	4.30	0.382^**^	0.394^**^	0.337^**^	0.303^**^	0.346^**^	0.891^**^	0.747^**^	1	
9Self-adjustment	39.26	4.74	0.405^**^	0.408^**^	0.373^**^	0.330^**^	0.348^**^	0.911^**^	0.772^**^	0.700^**^	1

^**^P < 0.01 (two-tailed)

Taking SRL as the dependent variable, we used forward stepwise regression to screen variables that had an impact on the SRL, such as specialty, score ranking, educational system, scholarship, whether be taught by a tutor in middle school or not, gender, father’s educational background, monthly family income, metacognitive strategy, resource management strategy, and cognitive strategy, which indicated a meaningful relationship with the SRL (*F* = 29.61, *p* < 0.01; *R*^2^ = 0.324, Adjust-*R*^2^ = 0.313). Regression analysis was performed, and the results are presented in Table 4.

**Table 4:** The multiple liner regression analysis results for Self-regulated Learning

**Factor**	**Unstandardized** **coefficient**		**Standardized** **coefficient**		
	**B**	**SE**	**β**	**T**	**p**
(Constant)	90.939	2.437		37.316	0.000
Like specialty^a^	4.305	0.797	0.159	5.399	**0.000**
Score ranking = 1^b^	5.827	1.252	0.191	4.655	**0.000**
Educational system^c^	-5.412	1.185	-0.152	-4.568	**0.000**
Scholarship^d^	2.239	0.84	0.088	2.665	**0.008**
Whether be taught by a tutor in middle school or not^e^	2.340	0.778	0.087	3.009	**0.003**
Score ranking = 2^f^	2.596	0.951	0.103	2.729	**0.006**
Gender^g^	-1.690	0.89	-0.057	-1.899	0.058
The father’s educational background^h^	-3.116	1.233	-0.073	-2.526	**0.012**
The mother’s educational background^i^	-0.930	0.832	-0.032	-1.117	0.264
Grade^j^	1.412	0.937	0.052	1.507	0.132
Monthly family income^k^	-2.300	0.888	-0.075	-2.590	**0.010**
Metacognitive strategy	0.367	0.064	0.355	5.744	**0.000**
Resource management strategy	0.285	0.118	0.096	2.410	**0.016**
Cognitive strategy	-0.190	0.096	-0.112	-1.969	**0.049**

Statistically significant values were indicated in bold, *F* = 29.61, *p* < 0.01; *R*^2^ = 0.324, Adjust-*R*^2^ = 0.313

*B* Regression coefficient, *SE* Standardized error

^a^ Yes:( 1,0,0),commonly:( 0,1,0),no:( 0,0,1)

^b^ downstream = 1, midstream = 2, upstream = 3

^c^ 3 years = 1,4 years = 2,5 years = 3

^d^ yes:(1,0),no:(0,1)

^e^ yes:(1,0),no:(0,1)

^f^ downstream = 1, midstream = 2, upstream = 3

^g^ Female:(0,1),Male:(1,0)

^h^ Primary school = 1, Middle school = 2, High school = 3, University = 4, University above = 5

^i^ Primary school = 1, Middle school = 2, High school = 3, University = 4, University above = 5

^j^ 1st grade = 1,2nd grade = 2,3rd grade = 3,4th grade = 4

^k^ Below CNY 2000 = 1,CNY 2001–5000 = 2,CNY5001-8000 = 3,CNY8001-10,000 = 4,CNY 10,000 above = 5

## Discussion

This study described the status quo of SRL of clinical medicine, TCM, nursing undergraduate, and junior college students at TCM University, analyzed the factors that affect the SRL of students at a TCM University in China, and revealed that individual factors, social factors, and Learning strategies mastered by students could affect their SRL.

### What is the current situation of SRL for medical-related students in TCM colleges and universities?

The score for learning strategy of medical-related undergraduates who were studying at a TCM University was 160.16 ± 29.45, and the score of each dimension was above the midpoint (3 points). This showed that the learning strategy of undergraduates was good. Metacognition can promote the mastery of metacognition skills, and they are also the basic skills of learners in self-regulation, critical thinking, and lifelong learning. Metacognitive skills are generally used to monitor and understand, regulate reasoning, and solve problems [31]. The better the learners’ metacognitive learning strategies, the better they can plan their learning, monitor, and evaluate their knowledge, deepen their perception of learning materials, find and solve problems more efficiently, and have more responsibility for their education, thereby urging them to study further [32]. Many studies have shown that metacognitive learning strategies could predict students’ academic success effectively [33–36].

Students should learn to take responsibility for their learning to acquire SRL skills and, thus, the ability to master lifelong learning [37]. The total score of SRL was 115.16 ± 12.42 in this study, which was above the median. It indicated that medical-related undergraduate students in TCM universities had good SRL abilities. Some studies have found that demand produces motivation, and clinical medical practice can enhance the SRL ability of medical workers [38]. However, most of our research objects were undergraduate students lacking clinical practice. Studying medical science, which has many knowledge essentials to remember, is perceived to be difficult commonly. Owing to the tremendous pressure of learning medical science, they need to know clinical medicine and TCM knowledge, as well as the numerous curriculum subjects and tight review time at the end of term. Students should possess good SRL ability of their volitional control, self-improvement, and self-evaluation. This also reminds us that educators should grasp the emphasis on students’ learning content, and the awareness of learning value as the focus. It is necessary to improve the teaching on learning strategies, and further improve students’ SRL ability ultimately. As medical knowledge changes daily and grows, it is more important than ever for medical educators to ensure that students have the necessary SRL skills, in addition to teaching medical knowledge [39].

### What factors influence the SRL of medical-related students in TCM colleges and universities?

In the univariate analysis, the SRL of male students were higher than that of female students, which is different from the conclusion that there is no difference in gender and female is stronger than male [40, 41]. Considering that the male subjects of this study were mostly majoring in traditional Chinese medicine and clinical medicine, those who could pass the college entrance examination and enter into medical colleges were also good at learning than those majoring in nursing. It found that the SRL of the students majoring in traditional Chinese medicine and clinical medicine were higher than the nursing students generally in this study, which is similar to the result that the SRL of clinical medicine was higher than other majors (imaging, nursing) [42]. Many studies have found that medical students have better academic performance. According to Zimmerman [43], high academic achievement is positively correlated with the mastery of learning strategies closely. Therefore, we can infer that medical students have better learning strategies than other specialties. Similarly, this conclusion could also be deduced to nursing students and had been verified [44]. In this study, the students with three years educational system mainly came from the extended education nursing college, which was different from the 5-year medical undergraduate and 4-year nursing undergraduate significantly, which indicated that there might be a correlation between college entrance examination scores and SRL indirectly. The SRL of students who were liberal arts divided subjects before admission were lower than those candidates from science. It is supposed that liberal arts students were good at the memory, while science students were adept at logical reasoning. However, basic medical knowledge requires physics, chemistry, biochemistry, and other scientific knowledge to understand when entering the field of medical science, which leads to difficulties and burnout for those liberal arts students, which may be the reason for their low SRL of them. In this study, the SRL of the students who are only child was stronger than that of non-only child, which was inconsistent with the original inference of the researchers. Considering that the education problems in Chinese families are all for the purpose to cope with the college-entrance examination. Although he has low self-care ability being only one child, he may not be poor in learning. Students who had scholarships had strong SRL, which was consistent with the results of Liu [45] that learning strategies are positively correlated with SRL. It indicated that students with scholarships had strong learning ability and self-control ability, and the acquisition of scholarships could be a positive feedback of independent learning ability. The SRL of the medical students who were admitted by the second and third batches were higher than those admitted by the extended education nursing college obviously, indicating that the college-entrance examination has the ability to distinguish IQ, self-management ability, and SRL again. This study found that SRL increased with grades, considering that medical students cannot establish a connection between the knowledge and their future work when they learned basic courses in the early stage. They felt the responsibility of treating patients and their love for specialty immediately when clinical courses permeated into their study with the grades increasing, which led to the growth of their SRL. This study found a close correlation between family monthly income and SRL. The result is similar to Zhang’s study [40]. What is different from Zhang [40] is that the higher the educational background of parents, the higher the SRL. Highly educated parents pay more attention to their children’s education and management. On the one hand, they could become a role model for their children to imitate. On the other hand, they could also provide more help in the learning process. Parents can act as coaches or mentors, whose support may be a prerequisite for the SRL process to realize its full potential in the performance stage [40]. The SRL of students who were in student cadres was higher than those who weren’t, which verified the result of Gong [46], that is, students who are students/community cadres have better learning motivation, learning strategies, and SRL. The results of this study showed that those who had taught by a tutor in middle school had stronger SRL. Considering that many students had attended extra-curricular cram schools in high school in Zhejiang Province, they might not be taken into account every moment and everyone. However, it could strengthen learning management, teach them learning strategies, and prompt the improvement of SRL through the one-to-few tutoring method. The SRL of students with upstream score ranking was higher than the others dramatically, which was consistent with the outcome that students with better academic performance have higher SRL [47], and proved that there was a positive correlation between SRL and academic performance. It suggested that this kind of deep learning approach encouraged learners to use metacognitive abilities to self-regulate learning, and positively understood their learning [48, 49]. The achievement of good grades can stimulate the motivation and enthusiasm of students to study independently. The SRL of students who liked their specialty was higher than the others. Educators should strengthen medical students’ cognition of their specialty, and enhance their emotional strategies.

Many factors influence the academic success of medical students, such as previous educational achievements, individual differences in learning strategies adopted in the learning process, and even personality characteristics [50]. A study on the gross anatomy course of first-year medical students found that those who used cognitive strategies that reinforce meanings, concepts, or generalizations and techniques that promote critical thinking, such as teacher/student questioning and peer teaching, had better academic performance [51]. Our correlation and regression analyses showed that learning strategies were positively correlated with SRL, which was similar to the outcomes of Zhang [52] on nursing students. These results showed that whether they liked their specialty, educational system, score ranking, scholarship, whether they were taught by a tutor in middle school or not, the father’s educational background, monthly family incomes, metacognitive strategy, resource management strategy, and cognitive strategy could all enter into the regression equation. They can predict a 32% variation in the SRL ability jointly. The standardized regression coefficients of metacognitive strategies, resource management strategies, and cognitive strategies were related to SRL observably. Therefore, it is more important to teach the content and methods of learning strategies, as is professional knowledge for students regardless of a TCM, nursing, or clinical medicine specialty.

### How can the SRL of medical-related students in TCM colleges and universities be improved?

SRL is an essential strategy for the continuous professional development of medical students. Medical education should not only teach them relevant knowledge but also teach the ability of SRL. According to Bandura’s triadic model of social cognition [53] (person-environment–behavior), Zimmerman [54] divided SRL into three phases: the forethought phase, the performance or volitional control phase, and the self-reflection phase. In the forethought phase, students analyze tasks, set goals, plan how to achieve those goals, and some motivational beliefs motivate this process and influence the activation of learning strategies. In the performance or volitional control phase, students perform the task while monitoring their progress, and using multiple self-control strategies to keep themselves cognitively engaged and motivated to complete the job. In the self-reflection phase, students assess how they performed the job and make attributions of success or failure. We made the following reflected on improving the SRL of medical-related students in TCM universities.

Firstly, improving students’ learning motivation is essential in enhancing SRL. In social cognition theory, Bandura believed that human behaviour and reason are influenced mutually, and people’s prediction of possible results of behaviors plays a crucial role in learning [53]. The teachers could provide more opportunities for students or immerse them in the clinical environment so that they can recognize what their responsibilities are in the clinical world. Demand-driven methods can improve the goal-oriented motivation of students in learning analysis, such as providing learning tasks with appropriate difficulty, emphasizing the necessity of lifelong learning in medical-related specialties, explaining the employment pressure caused by the epidemic, and the need for the postgraduate entrance examinations, etc. To change the concept of students from “I was asked to learn” to “I want to learn.” Meanwhile, various teaching methods can stimulate students’ learning motivation. Teachers should also use effective methods to reform or create Golden Lessons(first-class undergraduate educational courses proposed by the Ministry of Education of China, which are highly advanced, innovative, and challenging to improve students’ interests in learning, such as case teaching, scaffolding, Problem-Based Learning, and Case-Based Learning. For example, the combination of online game learning and observation-summary-question strategy not only improved the learning interest of nursing students, but also improved their nursing operation skills [55]. Virtual Reality experiential education and training can also improve the learning motivation of students successfully [56, 57]. In addition, merging Problem-Based Learning with Emulation-Based Learning in the undergraduate medical curriculum can promote students’ lifelong learning [58]. Secondly, according to Zimmerman’s SRL learning model theory [54], the learning strategies run through the whole theory, which is one of the indispensable conditions for promoting the improvement of SRL. Therefore, some learning strategy frameworks and training planning methods developed in recent years have also achieved results to increase students’ knowledge of effective learning strategies, and support their use of them ultimately [59–62]. For example, the Study-Smart program can improve the metacognitive knowledge of pharmaceutical students and enhance the use of effective learning strategies [62]. In the teaching process of professional courses, teachers developed teaching of Professional-Infiltration learning strategies, which also improved the score of students’ professional courses [63]. In terms of learning settings, the teachers should give students explicit learning content and teaching objectives whenever they teach a new course, and summarize the key points of the course before the end of the class. Moreover, specific learning strategies should also be taught, such as teaching students to plan, monitor, and regulate their learning behavior, training them to restate knowledge, refine and organize them. At the same time, teaching them how to manage the learning time and environment, and asking for help during difficulties, such as Mining Students’ Think-aloud Protocols, is applied to evaluate the SRL time [64]. Meanwhile, students should be taught to regulate themselves in learning. Example teaching method is a better self-regulation strategy, self-evaluation of learning content, methods, and effects should be carried out. Self-reward and self-reinforcement strategies should be adopted when they achieve phased learning success. Thirdly, self-reflection is also a necessary way to improve the SRL. Educators should cultivate students’ self-confidence, challengeable ability, and tolerance to cope with setbacks, assess and give feedback timely on students’ learning behaviors and outcomes, encourage students to think about the reasons for failure, and summarize the successful experience. Reflective evaluation intervention [65] also contributes to the improvement of SRL. Fourthly, the environment also plays an important role in stimulating SRL [54]. Studies have found that the clinical learning environment is conducive to the change of SRL [66]. Students who have learned PBL courses will have a higher level of SRL when they transition to the clinical learning stage [67], and experienced students are more likely to create their learning objectives [68]. For cultivating talents in traditional Chinese medicine, traditional Chinese medical classics are the foundation, education from a tutor is the key point, and clinical practice is the essential element [69]. The SRL also changes rapidly in the period of clinical practice after learning basic knowledge [67]. Therefore, it is necessary to arrange for the students of TCM to go to the outpatient clinic with the tutor to look, listen, question, and feel the pulse – four ways of diagnosis as soon as possible. Clinical medicine and nursing students are also arranged for clinical probation more and more to obtain the image understanding of medical knowledge. Empirical research found that the pre-internship model could improve the SRL of vocational nursing students whose educational system is five-year [70]. Fifthly, according to Hadwin’s theoretical model of Socially Shared Regulation of Learning (SSRL) [71], With “I” as the center of the SRL + “you” centered SRL + to “we” as the center of SRL, formed in the form of circulation between three parts of self-monitoring, mutual monitoring, and share three monitoring modes, such as monitoring [72]. The key issue is that it builds on and merges individual and social processes, which is explained by the activity of the social entity in a learning situation in SSRL [73]. Therefore, it is essential to get social support to improve students’ SRL. Teachers can assist the students in making short-term and long-term learning plans, and urge and help the students to solve problems. Group discussion or team courses could be carried out. Students can support and help each other to make progress together. Peer learning in clinical skills education could make students more responsible for their education, which should be supervised by faculty [74]. Let scholarship winners share their learning experiences to help others use some learning strategies according to different learning tasks selectively.

### Strengths and contributions of the current research

The study probed into the influence of personal factors (physical self, mental self, social self) and learning strategies on SRL deeply, analysed the differences of SRL among different medical-related students in a traditional Chinese medical university, which mainly focus on the education of traditional Chinese medicine, to provide a reference for exploring the teaching management of SRL under the background of the epidemic, and put forward suggestions from the forethought phase, performance or volitional control phase to self-reflection phase, respectively, to guide educators to improve the learning strategies and SRL of medical-related students.

### Limitations

This study explored the status quo of SRL and its influencing factors on medical-related students in a traditional medical university in China. However, there are some limitations in the study. Firstly, this study was a cross-sectional survey. We just only analyzed the effect of influence on SRL in terms of demographic characteristics and learning strategies, it is difficult to infer the causal relationship among these factors, and the impact of environmental and behavioral factors on SRL was not explored enough. Secondly, the results of the present study were related to the subjects in its sample, and thus, they could not be generalized. Therefore, the results should be interpreted with caution. Thirdly, the representatives of this study were recruited from a TCM university in Zhejiang Province. The interpretation of these results was likely to be limited because of the regional sampling limitations In addition, this study lacked empirical research to demonstrate the rationality of the inference and the effectiveness of the intervention. Consequently, large-scale samples and longitudinal intervention studies from different regions need to be designed to verify the results of this study.

## Conclusions

The study was conducted to explore the influencing factors of SRL of medical-related students in a traditional Chinese medical university from the perspective of self-concept. Self-concept factors, including physiological-self, psychological-self, social-self, and their influence on SRL from the standpoint of learning strategies throughout the theory of SRL. The SRL of students who like their specialty, the longer educational system, the top score ranking, getting the scholarship, having been taught by a tutor in middle school, the higher the father's educational background, the higher the monthly family income, and the better the metacognitive strategies, resource management strategies, and cognitive strategies were better. They are all predictors of SRL. Some suggestions were put forward to improve the SRL of medical-related students in TCM colleges. However, since most subjects were not in the internship, the study of environmental factors was insufficient. Therefore, the influence of environmental and behavioural factors on SRL needs to be studied further.

## Data Availability

The datasets used and/or analyzed during the current study are in Chinese and are available from the corresponding author on reasonable request but will require translation to English.
